# Ultrasensitive single-ion electrometry in a magnetic field gradient

**DOI:** 10.1038/s41567-025-02887-9

**Published:** 2025-06-11

**Authors:** F. Bonus, C. Knapp, C. H. Valahu, M. Mironiuc, S. Weidt, W. K. Hensinger

**Affiliations:** 1 Sussex Centre for Quantum Technologies, University of Sussex, Brighton, UK; 2 Department of Physics and Astronomy, University College London, London, UK; 3 Universal Quantum Ltd, Gemini House, Mill Green Business Estate, Haywards Heath, UK; 4 Present Address: School of Physics, University of Sydney, Camperdown, New South Wales Australia

**Keywords:** Quantum metrology, Quantum mechanics, Atomic and molecular physics, Quantum physics

## Abstract

Hyperfine energy levels in trapped ions offer long-lived spin states. In addition, the motion of these charged particles couples strongly to electric field perturbations. These characteristics make trapped ions attractive platforms for the quantum sensing of electric fields. However, the spin states do not exhibit a strong intrinsic coupling to electric fields, lim iting the achievable sensitivity. Here, we amplify the coupling between electric field perturbations and the spin states by using a static magnetic field gradient. Displacements of the trapped ion resulting from the applied electric field perturbations are thereby mapped to an instantaneous change in the energy-level splitting of the internal spin states. This gradient-mediated coupling of the electric field to the spin enables the use of well-established magnetometry protocols for electrometry, making it possible to achieve extremely sensitive measurements of d.c. and a.c. electric fields. We also employ a rotating-frame relaxometry technique and demonstrate the use of our quantum sensor as an electric field noise spectrum analyser. Finally, we describe a set of hardware modifications that are capable of achieving a further improvement in sensitivity by up to six orders of magnitude.

## Abstract (web summary)

Trapped ions are promising for electrometry but limited by their weak intrinsic spin coupling to electric fields. Now it is shown that using a magnetic field gradient enhances sensitivity and enables precise measurements across subhertz to kilohertz frequencies.

## Main

Precision measurements of electric fields and forces are used in a wide range of emergent applications in biological, biomedical and chemical research^1–4^, particle physics^4–6^, gravitational wave detection^7^, energy applications^8^ and communications^9,10^. Consequently, a variety of electrometers based on various quantum hardware platforms have been developed, including bulk^11^ and single^12^ nitrogen-vacancy (NV) centres, quantum dots^13^, Rydberg atoms^14–17^ and trapped ions in Penning and Paul traps^18–22^.

Existing quantum electrometers have demonstrated ultrasensitive electric field measurements. However, they are restricted to certain frequency bands, with few sensors being able to measure subkilohertz frequencies^23^. This is because commonly used electrometers rely on either near-resonant measurements of transitions within the quantum system^15,17,19,21,24^, or resonant pulse techniques on spin states using phase-coherent sensing protocols^11,12,20^. In the former, the measurement bandwidth is defined by the frequency of available transitions. In the latter, the lower cutoff frequency of the sensor is constrained by both the achievable coherence times and the coupling strength of the quantum states to the electric field perturbation, whereas the upper limit is restricted by the pulse duration of coherent operations on the spin states.

Access to the frequency band ranging from subhertz to several kilohertz could enable quantum electrometers to be used for a variety of other applications, including medical imaging techniques such as electrical impedance tomography^25^, microscopy^26^, meteorological applications such as the long-range geolocation of lightning^27^, as well as the study of atmospheric phenomena and space weather^28–30^. Geological prospecting techniques are another use case for a low-frequency sensor, where applications include the detection of a range of subterranean and submarine features^31,32^.

In this work, we describe a new quantum electric field sensor in which a magnetic field gradient is used to couple electric field signals to the energy-level separation between the spin states of a two-level system in a single trapped ion. We experimentally demonstrate d.c. and low-frequency a.c. electric field sensitivities that are unmatched by current state-of-the-art electrometers within our measurement bandwidth. We also demonstrate the versatility of our sensing scheme by employing a magnetometry technique to measure the electric field noise.

**Fig. 1:**
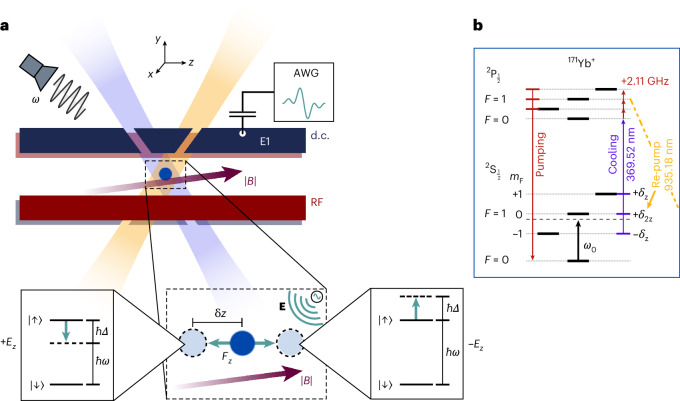
Electric field sensing with a trapped ion in a magnetic field gradient. **a**, Top, single ion confined in an RF Paul trap. Segmented d.c. electrodes (blue) provide confinement in the axial (*z*) direction. The RF electrodes (red) provide confinement in the radial (*x*, *y*) directions. A magnetic field gradient of ∂*B*/∂*z* = 22.41(1) T m^−1^ is applied along *z*. Doppler cooling and re-pump lasers at wavelengths of 369.52 and 935.18 nm, respectively, are indicated by the blue and orange beams. Transitions between the internal spin states are driven using an external microwave emitter. Electric field signals are applied to the ion through a d.c. end-cap electrode (E1) and are generated using an AWG that is capacitively coupled into the signal chain of E1. Bottom, zoom-in. An external electric field **E** applies a force **F** on the ion, resulting in a displacement *δz*. The transition frequency of the spin states is then shifted by *Δ* due to the magnetic field gradient. **b**, Simplified energy-level diagram of the ^171^Yb^+^ ion. Doppler cooling, optical pumping and state detection are carried out using the standard resonance fluorescence scheme described in ref. ^50^. Phase-coherent operations on the second-order magnetic field sensitive |*F* = 0, *m*_F_ = 0〉 to |*F* = 1, *m*_F_ = 0〉 transition and first-order sensitive |*F* = 0, *m*_F_ = 0〉 to |*F* = 1, *m*_F_ = +1〉 transition are driven by resonant microwave fields.

We consider a single ion with charge *q* confined in a radio-frequency (RF) Paul trap. A magnetic field gradient is applied at the position of the ion, as depicted in Fig. 1. A perturbation of the electric field *δE*(*t*) will alter the confining potential and exert a force *δ***F**(*t*) = *qδE*(*t*) on the ion. This force displaces the ion along the vector **r** = (*r*_x_, *r*_y_, *r*_z_) by an amount (Methods)

1 $$\delta {{{r}}}_{\rm{i}}(t)=\frac{{{q}}}{{{m}}{\nu }_{{{\rm{i}}}}^{2}}\delta {{{E}}}_{{{\rm{i}}}}({{t}}),$$

where *i* ∈ {*x*, *y*, *z*}, and *m* and *ν*_i_ are the mass of the ion and its vibrational frequency along the *i* axis respectively. The displacement *δr*_i_ of the trapped ion causes a change *Δ* in the transition frequency *ω* of its spin states due to the position-dependent Zeeman shift. The transduction parameter *γ*_i_ defines the susceptibility of the spin state transition frequency to changes in the electric field and is given by

2 $${\gamma }_{{{\rm{i}}}}=\frac{\partial \omega }{\partial {{{E}}}_{{{\rm{i}}}}}=\frac{\partial \omega }{\partial {{B}}}\frac{\partial {{B}}}{\partial {{{r}}}_{{{\rm{i}}}}}\frac{\partial {{{r}}}_{{\rm{i}}}}{\partial {{{E}}}_{{{\rm{i}}}}},$$

where ∂*ω*/∂*B* is the susceptibility of the transition frequency to changes in the magnetic field, ∂*B*/∂*r*_i_ is the strength of the magnetic field gradient along *r*_i_, and ∂*r*_i_/∂*E*_i_ = *q*/*mν*_i_^2^ is the change in position for a given change in the electric field at the ion. Equation (2) highlights the mechanism of our sensing scheme. The magnetic field gradient transforms electric fields into magnetic fields in the reference frame of the ion, which allows for the implementation of a wide range of magnetometry techniques for electrometry. From equation (2), we can see that stronger coupling is achieved by lowering the vibrational frequency of the ion, increasing the strength of the magnetic field gradient, using ions with a larger charge-to-mass ratio or by employing transitions with a higher susceptibility to magnetic fields. Electric field vector sensing is also in principle possible by tuning the confinement strength of the ion trap to maximize *γ*_i_ along one axis while suppressing it along the others.

All experimental demonstrations of our sensing scheme were conducted using a single ^171^Yb^+^ ion confined in a linear RF blade-trap with segmented d.c. electrodes^33^. A magnetic field gradient of ∂*B*/∂*z* = 22.41(1) T m^−1^ is generated along the axial (*z*) direction of the trap by a set of samarium-cobalt magnets. The magnetic field strength at the unperturbed ion position is *B*_0_ = 8.3767(4) G. Doppler cooling and re-pump lasers, with wavelengths of 369.52 and 935.18 nm respectively, are used to cool the ion to near the Doppler limit, whereas coherent operations on the spin states are realized by applying microwave fields using an external microwave emitter, as shown in Fig. 1. Further details of the experimental set-up and control techniques can be found in Methods. Electric field signals are generated by an arbitrary waveform generator (AWG) and injected onto one of the d.c. end-cap electrodes of the ion trap by capacitively coupling across a 220 pF capacitor (Methods). The applied electric field strength is characterized by a geometric factor *α*_i_ = ∂*E*_i_/∂*V*, which relates the electric field at the position of the ion to the d.c. voltage applied to the electrode. Stronger radial confinement (*ν*_x_/2π ≈ *ν*_y_/2π ≈ 1.5 MHz) suppresses coupling of the radial electric field components to the spin state transition frequency by a factor *γ*_z_/*γ*_x,y_ ≈ 180 (Methods). The subsequent experiments, therefore, measure solely the axial (*z*) component of the electric field. We find *α*_z_ = *α* = −95.64(4) (Methods), and we will drop the subscript from here on.

## a.c. and d.c. sensing

We use the |↓〉 = |*F* = 0, *m*_F_ = 0〉 and |↑〉 = |*F* = 1, *m*_F_ = 0〉 energy levels of the ^2^S_1/2_ hyperfine manifold of ^171^Yb^+^ for the measurements of a.c. and d.c. fields (Fig. 1b). The energy-level separation of the spin states is a function of the magnetic field at the ion, and is given by *ω* = *ω*_0_ + *δ*_2z_ where *ω*_0_/2π ≈ 12.64 GHz is the hyperfine splitting at zero magnetic field and *δ*_2z_/2π = 310.8*B*^2^ Hz (*B* in Gauss) is the second-order Zeeman splitting^34^. The vibrational frequency along *z* was measured to be *ν*_z_/2π = 161.191(8) kHz, from which we calculated the transduction parameter *γ* = 3,998(2) rad mV^−1^ (Methods).

**Fig. 2:**
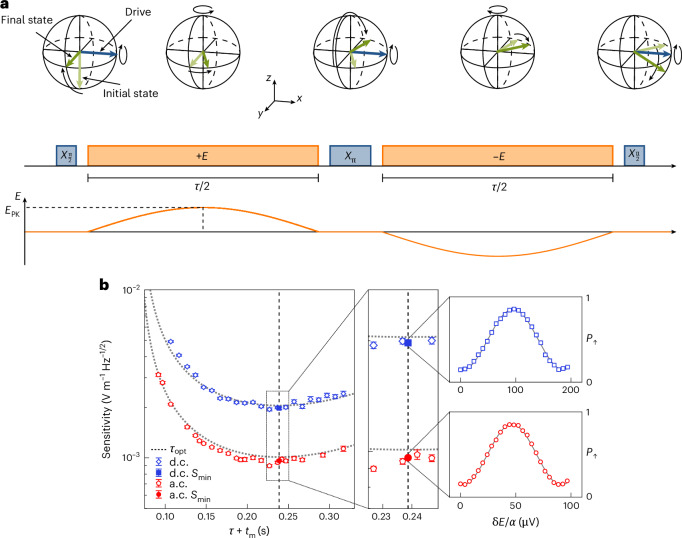
Measuring a.c. and d.c. sensitivities. **a**, Bloch sphere representation of the quantum state evolution (top), pulse sequence diagram (middle) and plot of the evolution of the electric field amplitude at the ion *E* for the a.c. sensing technique (bottom). Blue arrows and rectangles represent the microwave drive, and the orange rectangles and lines represent the interaction with the electric field. The initial and final spin states are shown in light and dark green, respectively. Each interaction period of duration *τ*/2 of the electric field features a half-oscillation of a signal with frequency *ω*_*ϵ*_/2 = 1/*τ*. *X*_π_ and *X*_π/2_ pulses are π and π/2 rotations around the *x* axis of the Bloch sphere induced by the microwave drive. **b**, Left, sensitivity of a.c. and d.c. sensing sequences against shot duration *τ* + *t*_m_ for evolution times ranging from *τ* = 25 to 250 ms, corresponding to signal frequencies *ω*_*ϵ*_/2π = 40 to 4 Hz. Centre, measurements of the sensitivity near the optimal evolution time *τ*_opt_ = 172(2) ms indicated by the squares and circles for d.c. and a.c., respectively, for 2,950 (d.c.) and 3,750 (a.c.) shots. Right, measured probability *P*_*↑*_ against the applied electrode voltage *δE*/*α* where *δE* = (2/π)*E*_PK_, for d.c. (a.c.) sensitivity measurements at *S*_min_ along with a least squares fit to a sine wave (solid grey) are shown in the upper (lower) plot. The dotted grey lines on the main plot are the theoretically expected curves for d.c. and a.c. sensing from equation (4). The error bars represent the 1*σ* confidence interval in the fitted fringes and the shot noise for the main plot and expanded plots, respectively.

The sensitivity to a.c. signals is characterized using a Hahn-echo-type sequence. The electric field signal is applied during the free precession time *τ*, as described in ref. ^35^ and illustrated in Fig. 2a. We apply an a.c. electric field with a frequency *ω*_*ϵ*_ = *τ*^−1^. The pulse sequence maps the electric field amplitude onto the probability of finding the spin in the |↑〉 state, *P*_*↑*_. The displacement of the ion in the magnetic field gradient results in an instantaneous field-induced detuning *Δ* of the two-level system transition frequency. A superposition of the spin states will, therefore, experience a phase shift of d*ϕ* = *Δ*(*t*) d*t*, where *Δ*(*t*) = *γδE*(*t*) is the detuning of the spin transition frequency. The total accumulated phase over the signal duration *τ* is $\phi =\int_{0}^{{\tau}/{2}}\varDelta (t)\,\mathrm{d}t-\int_{{\tau}/{2}}^{\tau}\varDelta (t)\,\mathrm{d}t$, which is a function of the electric field amplitude *δE*(*t*) and *τ*. The electric field amplitude is linearly increased for each interaction time *τ*, leading to sinusoidal oscillations of *P*_*↑*_. A linear least squares fit is used to fit an equation of the form ${{{P}}}_{\uparrow}=\frac{1}{2}+\frac{{{A}}}{2}\sin(\frac{2\uppi}{\kappa}{{E}})$ to the data. Here *A* is the fringe amplitude, *κ* is the electric field required to induce a 2π phase rotation of the spin and *E* is the electric field at the ion. We extract the resulting maximal derivative ∂*P*_↑_/∂*E* and use this to calculate the minimum detectable electric field:

3 $${{{E}}}_{\min }={\sigma }_{{\rm{tot}}}{\left(\frac{\partial {{{P}}}_{\uparrow }}{\partial {{E}}}\right)}^{-1},$$

where *σ*_tot_ is the total read-out uncertainty due to quantum projection noise (${\sigma }_{{\rm{quantum}}}^{2}$) and classical read-out noise (${\sigma }_{{\text{read-out}}}^{2}$) and is given by ${\sigma }_{{\rm{tot}}}^{2}={\sigma }_{{\rm{quantum}}}^{2}+{\sigma }_{{\text{read-out}}}^{2}\approx1/(4{{{C}}}^{2}{{N}})$ (ref. ^35^). Here, ${{C}}\approx 1/\sqrt{(1+4\eta )}$ is an overall read-out efficiency parameter^36^, *N* is the number of measurements of the spin state and *η* is the infidelity associated with state preparation and measurement (SPAM). We measure a SPAM infidelity of *η* = 1.8 × 10^−2^, resulting in *C* = 0.97. The sensitivity, defined as the minimum detectable signal measured over 1 s of averaging, is calculated as ${{S}}={{{E}}}_{\min }\sqrt{{{{t}}}_{\exp }}$. Here, ${{{t}}}_{\exp }={{N}}(\tau +{{{t}}}_{{\rm{m}}})$ is the total experimental duration, where *t*_m_ is the overhead associated with initialization, manipulation and read-out of the sensor. From ref. ^35^, the optimum sensitivity for a given evolution time *τ* is

4 $${{{S}}}_{\min }=\frac{\operatorname{e}^{\chi (\tau )}\sqrt{\tau +{{{t}}}_{{\rm{m}}}}}{\gamma {{C}}\tau },$$

where *χ*(*τ*) is the associated decoherence function of the two-level system. The measured sensitivity for each evolution time is shown in Fig. 2b. a.c. waveforms were applied across the capacitor for various evolution times. These waveforms were pre-compensated to account for the frequency-dependent phase offset induced by the capacitor (Methods).

Although d.c. signals cannot be injected across the input capacitor, the sensitivity of the sensor to d.c. electric fields is characterized by injecting a time-varying signal. We also employ a Hahn-echo-type sequence for d.c. sensing, where the interaction between the electric field and the sensor occurs only during the first half (*τ*/2) of the total free evolution time (Methods). The average electric field over the course of this half-oscillation is given by $\bar{{{E}}}=\frac{2}{\uppi }{{{E}}}_{{\rm{PK}}}$, where *E*_PK_ is the electric field amplitude. Correspondingly, the sensor accumulates the same amount of coherent phase *ϕ* as if it had evolved under a square d.c. pulse of amplitude ${{{E}}}_{{\rm{d.c.}}}=\bar{{{E}}}$.

The data shown in Fig. 2 are in good agreement with the theory, as plotted from equation (4). For a.c. sensing, a ~5% offset of the measured sensitivity relative to the theory is observed near *τ*_opt_. This is due to higher-frequency electric field components capacitively coupling onto the electrode, which could not be fully eliminated by the pre-compensation sequence.

The local minimum of the sensitivity *S*_min_ occurs at an optimal evolution time *τ*_opt_. This is because the phase accumulation induced by the electric field increases linearly with *τ* but is counteracted by the reduction in the fringe contrast of the quantum system due to decoherence, which followed a Gaussian functional form. *τ*_opt_ can therefore be determined from equation (4). Experimentally, we find the local minimum of the sensitivity to be at *τ*_opt_ = 172(2) ms for *t*_m_ = 66.839 ms and coherence time *T*_2_ = 304(3) ms (Methods). We measure a minimum a.c. sensitivity of ${{{S}}}_{\min }^{{\rm{a.c.}}}=960(10)\times 1{0}^{-6}\,{\rm{V}}\,{{\rm{m}}}^{-1}\,{{\rm{Hz}}}^{-{1}/{2}}$ at a signal frequency of ${\omega }_{\epsilon }={\tau }_{{\rm{opt}}}^{-1}=5.82\,{\rm{Hz}}$, and a minimum d.c. sensitivity of ${{{S}}}_{\min}^{{\rm{d.c.}}}=1.97(3)\times 1{0}^{-3}\,{\rm{V}}\,{{\rm{m}}}^{-1}\,{{\rm{Hz}}}^{-{1}/{2}}$.

**Fig. 3:**
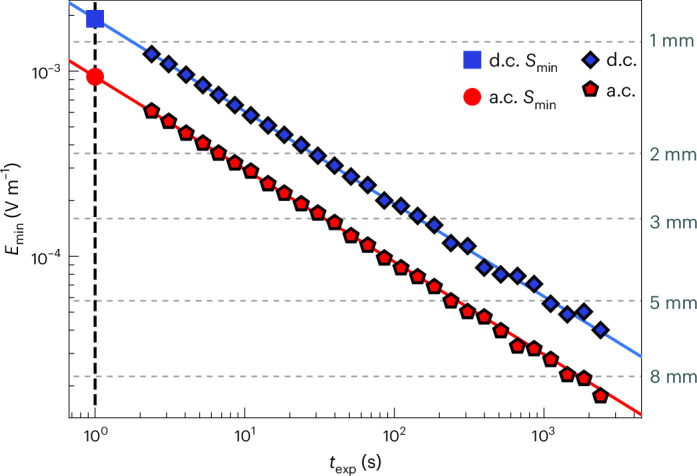
Minimum detectable signal against measurement time. Measured values of *E*_min_ at fixed measurement times for d.c. (a.c.) sensing shown in blue (red). The blue (red) lines show the theoretical dependence of *E*_min_, which is limited only by quantum projection noise. The value of *E*_min_ for a measurement time of 1 s (which defines the minimum sensitivity of the quantum sensor) is also shown (dashed black line). The classical read-out error is approximately equivalent for measurements on the |↓〉 and |↑〉 states, meaning that it does not contribute to the experimentally measured standard deviation shown in this figure. The dotted grey lines represent the magnitude of the electric field emanating from a single elementary charge at the indicated distance.

To determine if our quantum sensor is shot noise limited, *M* = 275,000 measurements (shots) are taken at the optimal evolution time *τ*_opt_ for both a.c. and d.c. signals. The electric field amplitude is set so that a measurement of the quantum system yields a probability *P*_*↑*_ = 0.5. The set of *M* shots is then subdivided into *k* = *M*/*N* sets of *N* shots. From this, we calculate *k* individual means, corresponding to the mean probability of each set of *N* shots. Using equation (3), we plot the minimum electric field, *E*_min_, calculated using the standard deviation of each set of *k* means (equation (3)), against *N* by varying the total measurement duration *t*_exp_, which is a function of *N* in Fig. 3. The measurement shows that the minimum detectable electric field follows a $1/\sqrt{{{{t}}}_{\exp }}$ dependence, which is consistent with a shot-noise-limited sensor. We find that for 1 s of integration time of an a.c. signal, the quantum electrometer is able to measure a minimum detectable electric field equivalent to an elementary charge at a distance of 1.225(6) mm.

## Rotating-frame relaxometry

**Fig. 4:**
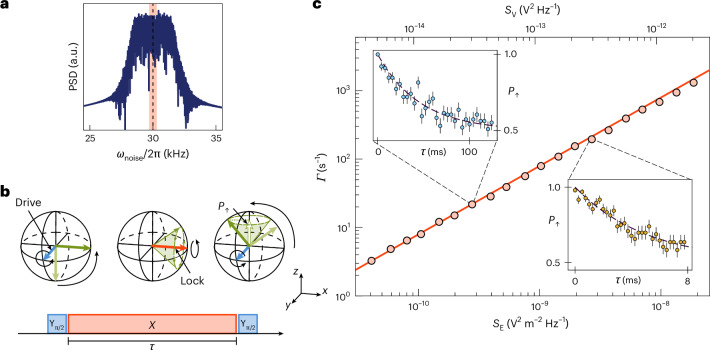
Rotating-frame relaxometry for electric field sensing. **a**, Periodogram of the applied noise. White noise with a bandwidth *B*/2π = 3 kHz centred around *ω*/2π = 30 kHz is applied to the system for various signal PSDs. The Rabi frequency *ω*_*ϵ*_ = 30.0 kHz of the spin-locking pulse is indicated by the black dashed line. The shaded region indicates the 1*σ* = ±300 Hz error of the Rabi frequency measurement. **b**, Pulse sequence diagram and associated Bloch sphere representation of the spin-locking sequence. A *Y*_π/2_ pulse aligns the spin state with the *x* axis. An *X* pulse with Rabi frequency *Ω*_X_ locks the state vector to the *x* axis. Resonant noise at the spin-locking Rabi frequency drives the |+〉 → |−〉 transition incoherently. A final *Y*_π/2_ pulse transfers the state population into the *σ*_z_ basis for read-out. The outer radius of the cone represents all possible alignments of the final state vector. The measured probability over many shots *P*_*↑*_ is represented by the projection of the vector onto the *z* axis (white vector). **c**, Measurement of the decay rate $\varGamma$ against the resonant electric field PSD *S*_E_ and voltage PSD *S*_V_ of the applied noise. Round markers indicate fits of probability measurements to exponential decay functions. Error bars are within the size of the marker. The solid line is given by equation (6). The left (right) inset shows measurements of the decay rate and the associated fit for a PSD of $S_{\rm{E}}$ = 2.770 × 10^−10^ V^2^ m^−2^Hz^−1^ ($S_{\rm{E}}$ = 2.689 × 10^−9^V^2^ m^−2^ Hz^−1^) resulting in a decay rate of $\varGamma$ = 22(1) s^−1^ ($\varGamma$ = 195(9) s^−1^). The error bars represent the 1*σ* confidence interval.

In the previous section, we have shown the measurement of d.c. signals and a.c. signals at well-defined frequencies and phases. Our sensor can, however, also be employed to measure stochastic signals with a discontinuous phase evolution over the measurement interval. We demonstrate this by using our sensing scheme to measure the power spectral densities (PSDs) of injected electric field noise. This is done using a spin-locking sequence. This technique is well established in magnetometry^37,38^. However, the gradient-mediated coupling of our scheme enables the implementation of spin-locking to measure the electric fields. The pulse sequence, outlined in Fig. 4b, begins by initializing the spin into the |+*X*〉 eigenstate. A resonant drive of the form (*ω*_*ε*_/2)*σ*_x_, with Rabi frequency *ω*_*ϵ*_, is applied parallel to the orientation of the spin state, locking the spin along the *x* axis of the Bloch sphere. The resonant interaction lifts the degeneracy of the |±*X*〉 eigenstates by an energy *ϵ* = *ℏω*_*ϵ*_, thereby making the two-level system sensitive only to *σ*_z_-type signals oscillating at angular frequency *ϵ*/*ℏ* = *ω*_*ϵ*_, effectively creating a quantum electric field noise spectrum analyser. In the presence of electric field noise, the resonant drive is applied for a duration *τ*, after which the spin state is mapped into the *σ*_z_ basis for detection. The measured probability follows an exponential decay over time of the form

5 $${P}_{\uparrow }=\frac{1}{2}\left(1+\operatorname{e}^{-\tau \varGamma }\right),$$

where $\varGamma$ is the decay rate of the system. The measured decay is a result of electric field noise at angular frequency *ω*_*ϵ*_ being transformed into *σ*_z_ noise on the spin states through the coupling induced by the magnetic field gradient. We define the PSD of the electric field noise at an arbitrary angular frequency *ω* as ${{{S}}}_{{{\rm{E}}}}(\omega)=\int_{-\infty}^{+\infty}\langle \delta {{E}}(0)\delta {{E}}({{t}})\rangle \operatorname{e}^{-{{i}}\omega {{t}}}\,\mathrm{d}t$. The corresponding PSD of the *σ*_z_ noise is then related to the PSD of the electric field noise by *S*_z_(*ω*_*ϵ*_) = *γ*^2^*S*_E_(*ω*_*ϵ*_), giving a spin-locking decay rate of^38^

6 $$\varGamma =\frac{1}{2}{{{S}}}_{{{\rm{z}}}}({\omega }_{\epsilon }).$$

Equations (5) and (6), therefore, make it possible to extract the PSD of the electric field noise at the angular frequency of the resonant drive *ω*_*ϵ*_.

To characterize our sensor experimentally, we capacitively inject electric field noise into the system for the duration of the spin-locking drive pulse. The waveform comprises white noise in a 3 kHz bandwidth centred around the resonant drive frequency *Ω*_X_/2π = 30.0(3) kHz, as illustrated in Fig. 4a.

For this experiment, we use the first-order magnetic field sensitive |↓〉 = |*F* = 0, *m*_F_ = 0〉 and |↑〉 = |*F* = 1, *m*_F_ = 1〉 spin states, where the transition frequency is *ω* = *ω*_0_ + *δ*_z_, and *δ*_z_/2π = 1.4 MHz G^−1^ is the first-order Zeeman shift. In addition, we set the axial secular frequency to *ν*_z_/2π = 264.79(1) kHz, from which we calculate a coupling strength of *γ* = 398.6(2) × 10^3^ radmV^−1^. We first verifiy the relation in equation (6) by characterizing the decay rate $\varGamma$ for various injected noise amplitudes (Fig. 4c). This is done by measuring *P*_*↑*_ as a function of the spin-locking drive duration *τ* and fitting the data to equation (5). We then characterize the minimum detectable signal, which is defined as the PSD of the electric field for which the signal-to-noise ratio is equal to 1. The signal-to-noise ratio is calculated by measuring the decay rate in the absence of injected noise. From this, we measure a decay rate $\varGamma_0$ = 0.49(4) s^−1^, corresponding to a minimum detectable signal of ${{{S}}}_{{\rm{E}}}^{\min }=6.2(5)\times 1{0}^{-12}\,{{\rm{V}}}^{2}\,{{\rm{m}}}^{-2}\,{{\rm{Hz}}}^{-1}$.

## Discussion

We describe a new quantum sensing technique for trapped ions in RF traps. A magnetic field gradient is used to couple displacements of the ion induced by the electric field to its spin state energy-level splitting, thus enabling the use of magnetometry protocols for electrometry. We demonstrated our scheme with a single trapped ^171^Yb^+^ ion by measuring the axial component of electric field signals emitted by an in-vacuum electrode. We measure a minimum a.c. sensitivity of ${{{S}}}_{\min }^{{\rm{a.c.}}}=960(10)\times 1{0}^{-6}\,{\rm{V}}\,{{\rm{m}}}^{-1}\,{{\rm{Hz}}}^{-{1}/{2}}$ for a signal frequency of *τ*^−1^ = 5.82 Hz and a minimum d.c. sensitivity of ${{{S}}}_{\min }^{{\rm{d.c.}}}=1.97(3)\times 1{0}^{-3}\,{\rm{V}}\,{{\rm{m}}}^{-1}\,{{\rm{Hz}}}^{-{1}/{2}}$. In addition, we employ a spin-locking sequence to measure stochastic signals with a discontinuous phase evolution over the measurement time. We determine a minimum detectable electric field PSD of *S*_E_(*ω*) = 6.2(5) × 10^−12^ V^2^ m^−2^ Hz^−1^ at a frequency of *ω*/2π = 30.0(3) kHz.

**Fig. 5:**
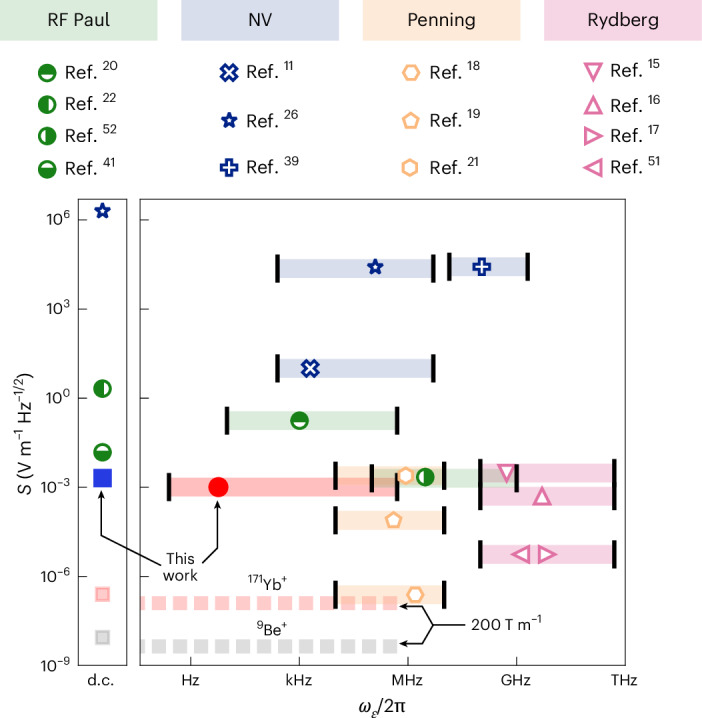
Comparison of the sensitivity and bandwidth of quantum electrometers. Comparison of electric field sensitivities and bandwidths of various quantum sensing hardware platforms. Markers show measured sensitivities as described in the corresponding reference, and shaded regions illustrate the approximate achievable bandwidths of each system. The blue square and red circle represent the measured sensitivities described in this work. The lower cutoff frequency of the experimental system is estimated for a coherence time limited by magnetic field noise at large *ν* corresponding to a cutoff frequency of 0.25 Hz. The dashed lines indicate the estimated achievable sensitivities for a system with *ν*/2π = 100 kHz and ∂*B*/∂*z* = 200 T m^−1^ using a first-order magnetic field sensitive state of ^171^Yb^+^ (light red) and ^9^Be^+^ (light grey). Penning^18,19,21^, Rydberg^15–17,51^, NV^11,26,39^ and Paul^20,22,41,52^.

Figure 5 compares the sensitivity and bandwidth of our scheme with those of current state-of-the-art quantum electrometers. Current quantum hardware platforms use a variety of measurement schemes for electrometry, resulting in a range of achievable bandwidths and measurable sensitivities. Single^26,39^ and bulk NV centres^11^ use resonant pulse schemes on their spin transition frequency and are able to operate at ambient conditions, allowing highly increased flexibility in sensor placement^3^. However, the coherence times and coupling strengths limit both the achievable sensitivities and the bandwidth. Rydberg atoms measure Stark shifts on internal transitions induced by near-resonant fields, enabling high-sensitivity electrometry in the 100 MHz to 500 GHz range^15,40^. Ion crystals in Penning traps are sensitive to electric fields at or near the motional resonances of the crystal, which are typically in the 50 kHz to 10 MHz range^18,19,21^. Rydberg and Penning trap architectures have also demonstrated electric field sensitivities below the standard quantum limit through entanglement-based schemes^15,18^. Finally, there exist a variety of sensors based on RF Paul traps, which implement both fluorescence-based schemes to measure d.c. electric fields^22,41,42^ and resonant pulse schemes for Doppler shift measurements^20^.

The achieved minimum sensitivities discussed in this work are unmatched by existing sensing hardware platforms across the measurement bandwidth of our sensor. Our sensing scheme can be used for highly sensitive electric field measurements in the d.c. and subhertz to ~500 kHz frequency range. The lower cutoff frequency is limited by the coherence time of the two-level system, whereas the upper cutoff frequency is a function of the maximal achievable Rabi frequency of the refocussing π pulses. Our experimentally measured optimal sensitivity is limited by both classical noise and hardware constraints specific to the experimental system. Voltage noise on the electrodes of the ion trap directly couples to the spin states, which limits the *T*_2_ coherence time. Previous measurements with our particular experimental set-up have shown that the coherence time of our system is dominated by voltage noise on the trapping electrodes and scales as *T*_2_ ∝ *ν*^4^(∂*B/*∂*z*)^−2^ (ref. ^43^). Equations (2) and (4) therefore indicate that the sensitivity in the current implementation of our electrometer is independent of both the secular frequency and the magnitude of the magnetic field gradient. However, this is not a universal scaling law, so that modifications to the hardware of the sensor would improve the measured *S*_min_ and further increase the bandwidth of the sensor. These modifications include reducing the PSD of the voltage noise on the electrodes, replacing the existing low-pass filter with one that has a larger roll-off rate and a lower cutoff frequency, or by using a voltage source that enables a different scaling of *T*_2_ with ∂*B/*∂*z* and *ν*_z_. Additionally, the time penalty associated with phase-matching electric field signals across the input capacitor leads to an increase in *t*_m_, which increases the minimum sensitivity achievable with the current experimental hardware (Methods). Using an in-vacuum antenna rather than a d.c. electrode as the electric field source would avoid the need for capacitive coupling of the electric field signals, thus leading to immediate improvements of *S*_min_.

The measured sensitivities could be improved through hardware modifications of the quantum sensor. Extending the coherence time by reducing the voltage noise on the electrodes, in combination with dynamical decoupling techniques, would enable the use of first-order magnetic field sensitive transitions as well as larger magnetic field gradients. Additionally, using a trapped ion with a larger charge-to-mass ratio, such as ^25^Mg^+^ or ^9^Be^+^, instead of ^171^Yb^+^ would improve the achievable sensitivities. For example, using the first-order magnetic field sensitive |*F* = 2, *m*_F_ = −2〉 to |*F* = 1, *m*_F_ = −1〉 transition in the S_1/2_ hyperfine manifold of ^9^Be^+^, in a system with ∂*B*/∂*z* = 200 T m^−1^, would result in a.c. sensitivities of <5 × 10^−9^ V m^−1^ Hz^−1/2^ for an evolution time of *τ*_opt_ = 170 ms (and *T*_2_ = 2*τ*_opt_). A further reduction in sensitivity by a factor of ${1}/{\sqrt{{{N}}}}$ could be achieved by simultaneously employing *N* trapped ions, which could be confined either in an array of trapping wells or as a large crystal in a single well. The size of larger crystals may be limited by a worsening of the sensitivity due to increased coupling to electric and magnetic field noise at large magnetic field, as a result of the second-order Zeeman shift, and the technical challenge of maintaining a large magnetic field gradient over the entire crystal length.

Miniaturization, portability and hardware complexity are also important considerations when deploying quantum sensors in the field^44^ and to ensure optimal positioning of the sensor relative to electric field sources. As the sensor presented in this work operates in-vacuum, sensor placement relative to a signal source may be more challenging for some applications. However, the development of compact ion-trapping systems is a well-established area of research, with substantial advances being made in vacuum system miniaturization^45,46^. Additionally, our scheme does not require cryogenic cooling of the hardware, which reduces the portability constraints.

In addition to improving sensitivities and portability, hardware modifications can broaden the range of applications of the sensor. A system that allows for independent tuning of the confinement strength along each axis of vibration can be used for the vector sensing of electric fields. Switchable static magnetic field gradients as described in ref. ^47^ could also be used to realize a hybrid magnetic field and electric field sensor, in which the sensor has an identical measurement bandwidth for both magnetic and electric fields. Furthermore, our electric field sensor is compatible with entanglement-enhanced sensing techniques. Static magnetic field gradient entanglement schemes for trapped ions using long-wavelength radiation^48,49^ can be implemented and could allow the sensor to reach sensitivities below the standard quantum limit.

## Methods

### Transduction parameter

We consider the dynamics of a string of *N* trapped ions perturbed by an external electric field, which results in a force *δF*_*j*_(*t*) = −*qδE*_*j*_(*t*) on ion *j*. Restricting ourselves to a single direction without loss of generality, the Lagrangian of this system is^53^

7 $$\begin{aligned}{{L}}&=\frac{{{m}}}{2}\left(\sum_{{{p}}=1}^{{{N}}}{(\dot{Q}_{p}({{t}}))}^{2} - {\nu}_{{{p}}}^{2}{{{{Q}}}_{{{p}}}}^{2}({{t}})\right)\\ &\quad{}+{{q}}{{{Q}}}_{{{p}}}({{t}})\sum_{{{j}}=1}^{{{N}}}{{{b}}}_{{{j}}}^{({{p}})}\delta {{{E}}}_{{{{j}}}}({{t}}),\end{aligned}$$

where *ν*_*p*_ are the normal mode frequencies and ${{{b}}}_{{{j}}}^{({{p}})}$ describes how strongly ion *j* couples to the mode *p*. The normal modes of motion *Q*_*p*_(*t*) are related to small displacements of the ion, *δr*(*t*) of equation (1), via:

8 $${{{Q}}}_{{{p}}}({{t}})=\sum_{{{j}}=1}^{{{N}}}{{{{b}}}_{{{j}}}}^{({{p}})}\delta {{r}}({{t}}).$$

The equation of motion of the *p*th normal mode is found from the Lagrangian using the relation

$$\frac{\rm{d}}{\rm{d}t}\left(\frac{\partial {{L}}}{\partial \dot{Q}_{p}(t)}\right)=\frac{\partial {{L}}}{\partial Q_{p}(t)},$$

resulting in

9 $${\ddot{{{Q}}}}_{{{p}}}({{t}})+{\nu}_{{{p}}}^{2}{{{Q}}}_{{{p}}}({{t}})=\frac{{{e}}}{{{m}}}\sum_{{{j}}=1}^{{{N}}}{{{b}}}_{{{j}}}^{({{p}})}\delta {{{E}}}_{{{j}}}({{t}}).$$

Without loss of generality, we restrict ourselves to a single-ion chain, *N* = 1, and consider the centre-of-mass motion along the *z* axis. After setting *p* = *z* and ${{{b}}}_{1}^{(1)}=1$, equation (9) becomes

10 $${\ddot{{{Q}}}}_{{\rm{z}}}({{t}})+{\nu}_{{\rm{z}}}^{2}{{{Q}}}_{{\rm{z}}}({{t}})=\frac{{{e}}}{{{m}}}\delta {{E}}({{t}}).$$

This corresponds to the equation of a driven harmonic oscillator. Taking the Fourier transform, equation (10) becomes

11 $${\hat{{{Q}}}}_{{{p}}}(\omega)=\frac{{{e}}}{{{m}}({\nu}_{{\rm{z}}}^{2}-{\omega}^{2})}\delta \hat{{{E}}}(\omega),$$

where $\hat{\cdot }$ denotes the Fourier transform. For *N* = 1 ion, Q_*p*_(*t*) = *δr*(*t*) and equation (11) becomes

12 $$\delta \hat{{{r}}}(\omega)=\frac{{{e}}}{{{m}}({\nu}_{{\rm{z}}}^{2}-{\omega}^{2})}\delta \hat{{{E}}}(\omega).$$

In the limit *ν*_z_ ≫ *ω*, equation (12) reduces to

13 $$\delta \hat{{{r}}}(\omega)=\frac{{{e}}}{{{m}}{\nu}_{{\rm{z}}}^{2}}\delta \hat{{{E}}}(\omega),$$

from which one can retrieve the expression of equation (1). From equation (12), we also find that the coupling of radial micromotion into the spin states is negligible. These oscillations occur at the RF trap frequency, *Ω*_RF_/2π = 19.22 MHz, and the resulting amplitude of the radial oscillation is negligible because *Ω*_RF_ ≫ *ν*_x,y_.

### Experimental set-up

Extended Data Fig. 5 shows a schematic of the experimental set-up used in this work. The ion trap was mounted inside a vacuum chamber maintained at an average pressure of 2.4 × 10^−11^ mbar. The ion is Doppler cooled using a 369.52 nm laser that is red-detuned from the ^2^S_1/2_ |*F* = 1〉 to the ^2^P_1/2_ |*F* = 0〉 transition. The laser beam is double-passed through an acousto-optic modulator to allow for fine frequency and amplitude control by a field-programmable gate array. An electro-acoustic modulator (EOM) is used to generate 2.11 GHz sidebands for state preparation. These sidebands allow the population to be driven into the ^2^P_1/2_ |*F* = 1〉 state via optical pumping, after which it decays into the |↓〉 = ^2^S_1/2_ |*F* = 0〉 ground state. The population that is off-resonantly driven into the ^2^S_1/2_ |*F* = 0〉 state during Doppler cooling is returned to the cooling cycle by continuously applied microwaves near 12.64 GHz. Population can also leak out of the Doppler cooling cycle by decaying into the ^2^D_3/2_ manifold, where a 935.18 nm re-pump laser applied on the ^2^D_3/2_ to ^3^D[3/2]_1/2_ transition returns population to the ^2^S_1/2_ |*F* = 1〉 state. The re-pump laser is also modulated by an EOM at 3.07 GHz to improve the re-pumping efficiency. Microwaves are generated by a vector signal generator (Keysight E8267D PSG), which produces a carrier signal of 12.54 GHz. This is then mixed with RF pulses near 100 MHz generated by a two-channel AWG (Keysight M8190A), which is then amplified and emitted by an external microwave emitter to allow for coherent manipulation of the spin state. The spin state is measured using a state-dependent fluorescence scheme as described in ref. ^50^. The average SPAM error was found to be *η* = 1.8 × 10^−2^. The voltage signals used to measure the a.c. and d.c. sensitivities are applied directly to the capacitor from the second channel of the AWG. To measure the electric field noise, a white-noise waveform is generated using a separate AWG (Agilent 33522A). The white-noise signal is attenuated by two 30 dB RF attenuators, and its output controlled with an external RF switch.

### Gradient measurement

**Extended Data Fig. 1:**
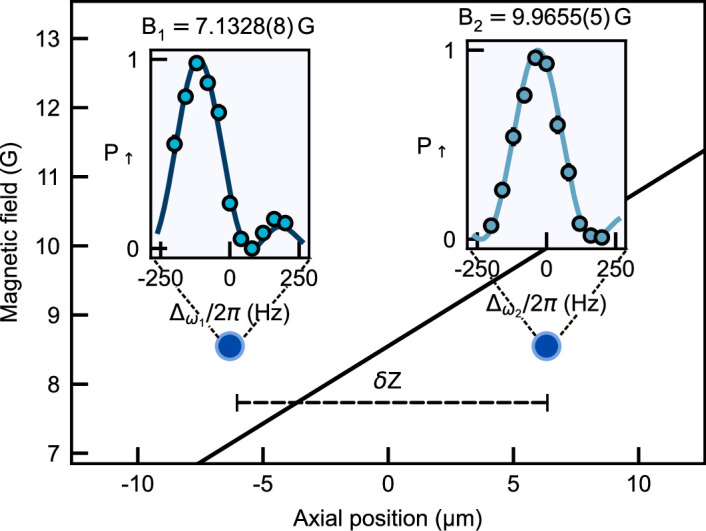
Measurement of the magnetic field gradient. The blue circles indicate the axial position of two co-trapped ^171^Yb^+^ ions. The axial (z) separation of the ions is calculated from spectroscopy measurements of their motional frequency, giving 12.64(1) μm, and is symmetric about the single ion equilibrium position in the axial direction of the trap. The insets show measurements and least-squares fits of the spin state transition frequency of each ion. The horizontal axis of the insets indicates the detuning of the applied microwave pulse, relative to probing frequencies near 12.64 GHz corresponding to the spin transition of ion 1, *ω*_1_ (left inset), and ion 2, *ω*_2_ (right inset). Inset titles are the values of the magnetic field magnitude calculated from the measured spin transition frequency of each ion. The magnetic field gradient is calculated using equation (14). The black line is a plot of magnetic field strength against axial position based on the measured magnetic field gradient. The error bars represent the 1*σ* confidence interval.

The strength of the magnetic field gradient along the axial direction was calculated by measuring the transition frequencies of two co-trapped ^171^Yb^+^ ions. As the splitting of the ^171^Yb^+^ spin states is dependent on the strength of the magnetic field at the position of the ion, the magnetic field gradient in the axial direction is given by

14 $$\frac{\partial {{B}}}{\partial {{z}}}=\frac{{{{B}}}_{2}-{{{B}}}_{1}}{\delta {{Z}}},$$

where *B*_1_ and *B*_2_ are the magnetic field strengths at the location of each ion, and *δZ* is the ion separation (Extended Data Fig. 1). The ion separation is a result of the mutual Coulomb repulsion between the ions and the oppositely acting axial confinement force. *δZ* is given by^53^

15 $$\delta {{Z}}={\left(\frac{{e}^{2}}{4\uppi {\epsilon}_{0}{{m}}{\nu}_{\rm{z}}^{2}}\right)}^{1/3}\frac{2.018}{{{{N}}}^{0.559}},$$

where *ν*_z_ is the axial vibrational centre-of-mass frequency, *m* is the mass of a single charged particle and *N* is the number of ions in the crystal. We measured *ν*_z_/2π = 161.191(8) kHz via the ‘tickling’ method. An a.c. electric field was applied to the trap using an external RF coil, which excites the axial motion of the ion crystal when the applied frequency is resonant with the axial vibrational frequency, leading to a measurable decrease in ion fluorescence due to the Doppler shift. We then compute *δZ* = 12.64(1) μm from equation (15).

The magnetic field at each ion was calculated by measuring the magnetic field-dependent transition frequency of each ion, as shown in the inset plots of Extended Data Fig. 1. From these measurements, *B*_1_ = 7.1328(8) G and *B*_2_ = 9.9655(5) G. Finally, from equation (14), the magnetic field gradient strength was ∂*B*/∂*z* = 22.41(1) T m^−1^.

### Calibrating *α* and *γ*

**Extended Data Fig. 2:**
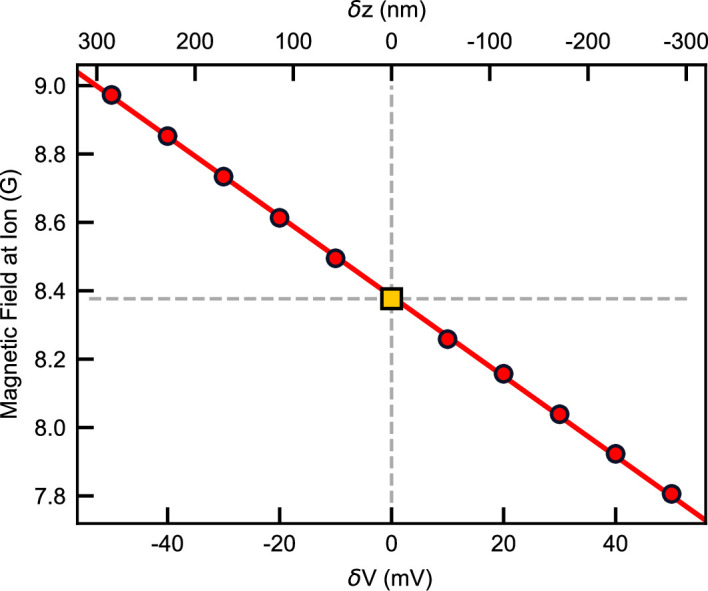
Calibrating the geometric factor. Measurement of the shift in resonance frequency of the two-level system after applying a static voltage offset *δV* to the E1 electrode. The red circles indicate the magnetic field determined from spin state transition frequency measurements at different values of *δV*, whilst the yellow square represents the magnetic field corresponding to a measurement of the unperturbed (*δV* = 0) transition frequency. The data are fitted to a straight line using a least-squares fit, shown in red. The top axis shows the axial displacement of the ion for a given *δV*, which is calculated using the previously measured value of *α*. Error bars are within marker size and represent the 1*σ* confidence interval.

The geometric factor of an electrode, *α*, relates the electric field at the position of the ion to the voltage applied to the electrode, and is defined as

16 $$\alpha =\frac{\partial {{E}}}{\partial {{V}}}=\frac{\partial \omega}{\partial {{V}}}\frac{\partial {{E}}}{\partial {{z}}}{\left(\frac{\partial {{B}}}{\partial {{z}}}\frac{\partial \omega}{\partial {{B}}}\right)}^{-1},$$

where ∂*E*/∂*z* = *mν*_z_^2^/*e*. We calibrate *α* by first measuring the change in magnetic field at the ion due to a change in the voltage applied to the E1 electrode (∂*B*/∂*V*) using the second-order sensitive spin state transition frequency and *ν*_z_/2π = 161.191(8) kHz (Extended Data Fig. 2). The measurement was performed with a single ^171^Yb^+^ ion by applying a voltage *V*_0_+*δV* to the electrode, where *V*_0_ = 1.75 V is the static voltage contributing to the axial confining potential and *δV* is an offset that is varied from −50 to +50 mV. We extract the value of ∂*B*/∂*V* from a least squares fit to a straight line of the magnetic field measurements for each voltage offset. From this, we then determine

$$\frac{\partial \omega}{\partial {{V}}}=\frac{\partial {{B}}}{\partial {{V}}}\frac{\partial \omega}{\partial {{B}}}=-382\times 1{0}^{3}\,{\rm{rad}}\,{{\rm{V}}}^{-1}.$$

The geometric factor is then calculated from equation (16), giving *α* = −95.64(4) m^−1^.

The transduction parameter is found using

$$\gamma =\frac{1}{\alpha }\frac{\partial \omega }{\partial {{V}}}=\left(\frac{\partial {{V}}}{\partial {{E}}}\frac{\partial \omega }{\partial {{V}}}\right).$$

For the second-order magnetic field sensitive transition, we measure *γ* = 3,998(2) rad m V^−1^.

Our scheme measures the electric field component along the *z* axis, as the sensitivities to electric fields in the *x* and *y* axes are negligible. To see this, we calculate the ratio between the transduction parameter in the *z* direction, *γ* = *γ*_z_, and the transduction parameter in the *x* and *y* directions, *γ*_x,y_, using equation (2). The magnetic field gradient along the *z* axis was measured to be ∂*B*/∂*z* = 22.41(1) T m^−1^, whereas the gradient along the *x* and *y* axes was estimated through numerical simulations to be ∂*B*/∂*r*_x,y_ ≈ 11 T m^−1^. With the motional frequencies *ν*_z_/2π = 161.191(8) kHz and *ν*_x,y_/2π ≈ 1.5 MHz, the ratio of the transduction parameters is

$${\gamma}_{{\rm{z}}}/{\gamma}_{{\rm{x}},{\rm{y}}}=\frac{\partial {{B}}}{\partial {{z}}}\frac{\partial {{z}}}{\partial {{{E}}}_{{\rm{z}}}}\left/\frac{\partial {{B}}}{\partial {{{r}}}_{{{\rm{x}}},{\rm{y}}}}\frac{\partial {{{r}}}_{{{\rm{x}}},{\rm{y}}}}{\partial {{{E}}}_{{{\rm{x}}},{\rm{y}}}}\approx 180,\right.$$

which indicates that the sensitivity to electric fields in the radial direction is over two orders of magnitude weaker.

### Electric field sensing protocol

**Extended Data Fig. 3:**
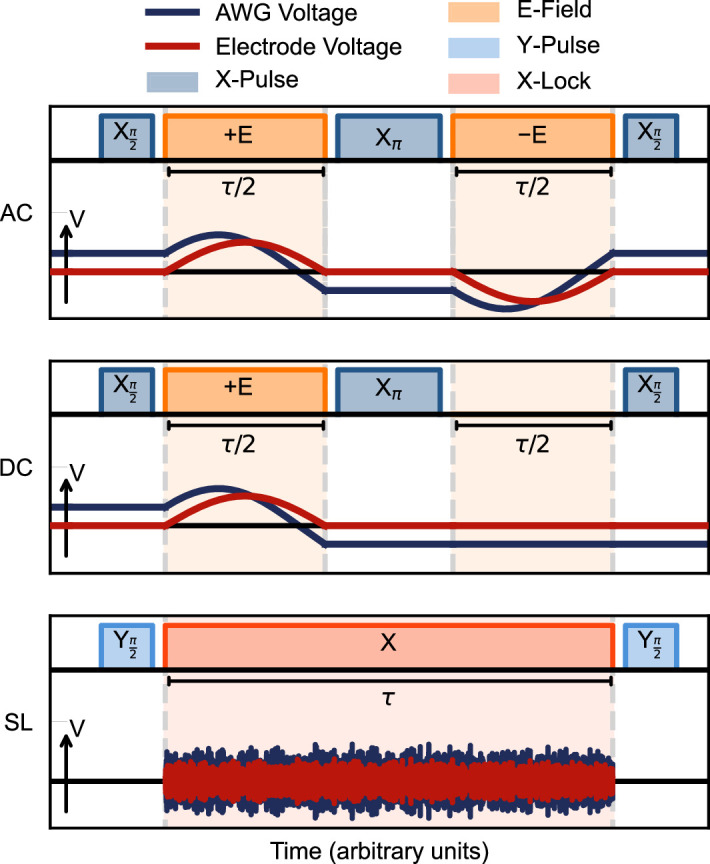
AC, DC and spin-locking (SL) pulse sequence diagram and time evolution of input signal. Blue lines represent the AWG voltage output, whilst red lines show the voltage evolution on the in-vacuum electrode. Note that the voltage on the electrode is attenuated and phase-offset relative to the AWG voltage. The AC sensing technique is characterized by applying a full oscillation of an AC signal at frequency $\frac{1}{\tau }$ onto the electrode. Sensitivity to DC signals is characterized by applying a half-oscillation of an AC signal at frequency $\frac{1}{\tau }$ onto the E1 electrode of the ion trap. For spin-locking, noise resonant with the spin-locking Rabi frequency is applied onto the electrode. The input signal exhibits a frequency dependent phase shift and a frequency dependent attenuation across the input capacitor. For AC and DC sensing the offsets are pre-compensated, as can be seen by the dark-blue line. For spin-locking a continuous signal is switched into, thus pre-compensation is not applied.

For the sensing of a.c. fields, we follow the pulse sequence protocol outlined in ref. ^35^ and illustrated in Extended Data Fig. 3. The a.c. sensing sequence is realized by first initializing the two-level system into the $\vert +\rangle =({1}/{\sqrt{2}})(\vert \downarrow \rangle +\vert \uparrow \rangle )$ state using a π/2 pulse. The superposition state then evolves under an electric field perturbation for a time *τ*/2. A π pulse reorients the spin along the equator of the Bloch sphere, before the quantum state again evolves under the electric field perturbation for a time *τ*/2. A final π/2 pulse mapps the state population into the *σ*_z_ basis for detection. Using this pulse sequence, the sensitivity of the spin state transition frequency is maximized for a.c. signals oscillating at a frequency of *τ*^−1^.

The d.c. sensing experiments also use a Hahn echo type pulse sequence, whose benefits are twofold. First, the coherence time of the sensor is greatly extended when compared to that of the Ramsey-type sequence, which allows for increased sensitivities. Second, the refocusing π pulse also compensates for detuning errors in the microwave pulses. The pulse sequence is illustrated in Extended Data Fig. 3, and begins with a π/2 pulse to initialize the spin into the $\vert +\rangle =({1}/{\sqrt{2}})(\vert \downarrow \rangle +\vert \uparrow \rangle )$ state. d.c. signals cannot be applied through a capacitor. The low-pass filter signal chain of the d.c. electrode is also not suitable for fast application of d.c. square pulses during the sensing pulse sequence, as the low-pass filter would significantly attenuate and distort the signal. Therefore, to quantify the sensor’s response to d.c. signals, we apply an a.c. signal of frequency *τ*^−1^ for the duration of the first *τ*/2 delay time. This corresponded to an equivalent d.c. voltage on the electrode of *V*_d.c._ = (2/π)*V*_PK_, where *V*_PK_ is the amplitude of the applied signal. Here, (2/π)*V*_PK_ is the average voltage over the half-oscillation of the a.c. waveform. The applied time-varying pulse therefore causes the spin state to accumulate the same amount of phase *ϕ* as a square d.c. pulse of amplitude (2/π)*V*_PK_ applied for a duration *τ*/2 based on the equation relating phase accumulation to the detuning of the spin transition: $\phi =\int_{0}^{{\tau}/{2}}\gamma \alpha \delta {{V}}({{t}})\,\mathrm{d}t$. The refocusing π pulse is then applied, followed by the second *τ*/2 delay time, during which no other voltage signals are applied to the electrode, followed by a final π/2 pulse.

In addition to the electric field interaction time *τ*, the second relevant time parameter from equation (4) is *t*_m_, which breaks down as follows for our experimental implementation: (1) d.c. offset application delay time, 50 ms (see next section), (2) Doppler cooling and detection, 14.599 ms, (3) state preparation and microwave pulses, 2.155 ms and (4) data processing and field-programmable gate array delays, 85 μs. The total *t*_m_ = 66.839 ms.

### Capacitive coupling of a.c. signals

Due to the absence of an in-vacuum antenna, the electric field signals measured by the trapped ion were emitted from an in-vacuum end-cap electrode, which also generated a d.c. confinement electric field. Voltage waveforms were generated by an AWG and capacitively coupled onto the electrode across a 220 pF capacitor. Due to their frequency-dependent impedance, capacitors act as high-pass filters, thereby attenuating the lower-frequency signals more strongly. The fixed response time of a capacitor will also shift the phase of a.c. signals that are applied across it. This shift in phase of the a.c. signal can, if unaccounted for, affect the total coherent phase *ϕ* that is accumulated by the spin states. To achieve an optimal measurement of the sensitivity of our experimental system, it is necessary for the electric field signal at the ion to be in phase with the Hahn-echo sensing pulse sequence. This is because *ϕ* is the difference between the coherent phase accrued during the first and second interaction times *τ*/2. An electric field signal that is not in phase with the Hahn-echo sequence will, therefore, reduce the measured sensitivity. References ^11^ and ^35^ provide further information about this effect.

We measure the phase shift on signals applied across the capacitor for the span of frequencies used in the a.c. and d.c. sensing experiments using an oscilloscope. Based on these measurements, we then pre-compensate the signal applied across the capacitor by applying an inverse phase shift, negating the effect of the capacitor on the phase of the voltage waveform. This ensures that the voltage on the electrode and, therefore, the electric field signal at the ion, are in phase with the Hahn-echo sequence.

Shifting the phase of the voltage waveform introduces a discontinuity into the signal. This manifests as a sudden change in the voltage across the capacitor from 0 to *V*_*Φ*_ = *V*_A_ sin *Φ*, where *Φ* is the phase of the a.c. voltage signal. Given that the current across a capacitor is defined as *I* = *C* d*V*/d*t*, where *C* is the capacitance of the capacitor, the high rate of change of voltage induces a large current flow across the capacitor, which introduces additional coherent phase offsets of the superposition state. To suppress this unwanted perturbation, we apply a d.c. voltage offset of *V*_*Φ*_ into the capacitor in the time before the initialization of the |+〉 state, which minimized the sudden voltage spike across the capacitor from the phase-shifted a.c. voltage waveform. To ensure that the sensor reaches a steady state before the application of the a.c. electric field signal, an extra 50 ms delay is added between the application time of the d.c. offset and the first resonant microwave pulse. This made up most of the *t*_m_ time, which was broken down in the previous section. The pre-compensation technique for the a.c. and d.c. sensing pulse sequences is visualized in Extended Data Fig. 3, which illustrates both the AWG and in-vacuum electrode voltage evolution throughout the experimental pulse sequence.

We also measure the frequency-dependent attenuation of the capacitor using an oscilloscope. We determine the transfer function of the capacitor by fitting a Butterworth high-pass filter function to these data. We then find the total attenuation of the electric field signal for a given frequency *τ*^−1^.

### Determination of the coherence time

**Extended Data Fig. 4:**
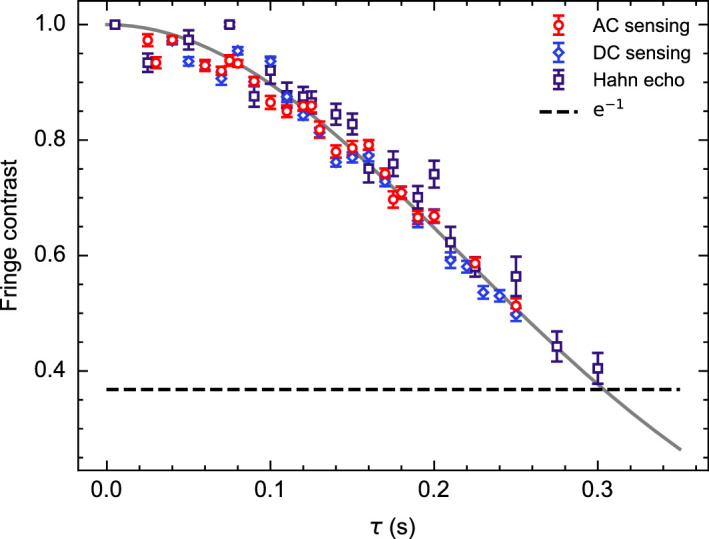
Coherence time measurement of the second order sensitive clock states. The spin states used for the experimental demonstration of AC and DC sensing are defined by the |↓〉 = |*F* = 0, *m*_F_ = 0〉 and |↑〉 = |*F* = 1, *m*_F_ = 0〉 energy levels. The fringe contrasts associated with each of the AC sensing, DC sensing, and Hahn-echo experiments are shown for a range of free evolution times *τ*. The black dashed line indicates the 1/e threshold. The grey line is a least squares fit of these measurements to a Gaussian decay function, corresponding to a coherence time of T_2_ = 304(3) ms. The error bars represent the 1*σ* confidence intervals in the fits to the fringe contrasts.

**Extended Data Fig. 5:**
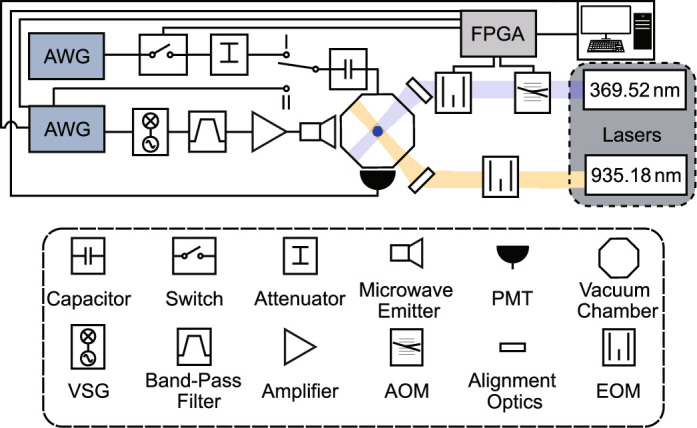
Experimental Setup. Electric field sensing configurations, coherent control, optical and electric field signal chains for the operation of the quantum sensor. Coherent control is achieved using triggered microwave pulses generated by amplitude modulation of an RF signal from a two-channel AWG with a microwave carrier using a VSG. The microwave tone is amplified and emitted into the vacuum chamber using a microwave horn. The second channel of this AWG provides the electric field signals for AC and DC sensing. These signals are synchronously coupled into the quantum sensor in configuration II. Configuration I shows the setup for rotating frame relaxometry. Here, a signal is continuously output using a second AWG. Interaction with the spin state is toggled using an RF switch. The signal is then attenuated and capacitively coupled onto the electrode. Doppler cooling, optical pumping and state detection of the ion are achieved by modulating a 369.52 nm laser beam using an acousto-optic modulator (AOM) and an electro-optic modulator (EOM). An EOM in the 935.18 nm beam allows for efficient repumping. The photo-multiplier tube (PMT) is used for fluorescence detection of the spin state.

We measure the coherence time of the two-level system using a Hahn-echo experiment. The spin is initialized in the |↓〉 state, after which a π/2 pulse rotates the spin into the |+*X*〉 eigenstate. A refocusing π pulse is applied between the two free evolution periods of duration τ/2. A final π/2 pulse maps the state into the *σ*_z_ basis for detection. Varying the phase of the final pulse from −2π to 2π results in sinusoidal fringes in the probability of measuring |↑〉. As the free evolution time is increased, decoherence leads to a reduction in the amplitude of these fringes. The coherence time *T*_2_ is given by the point at which the fringe contrast reaches *e*^−1^. As the a.c. and d.c. sensing experiments were also based on the Hahn-echo sequence, the fringe amplitudes from these experiments can also be used for the coherence time measurement. The fringe amplitudes in these three experiments are shown against the free evolution time in Extended Data Fig. 4. These data are aggregated and fitted to a Gaussian decay function of the form *χ*^−1^(*t*) = exp(−*t*^2^/*T*_2_^2^) using a least squares fit, yielding a coherence time of *T*_2_ = 304(3) ms.

## Online content

Any methods, additional references, Nature Portfolio reporting summaries, source data, extended data, supplementary information, acknowledgements, peer review information; details of author contributions and competing interests; and statements of data and code availability are available at 10.1038/s41567-025-02887-9.

## Data Availability

The data that support the findings of the study are available from the corresponding author upon reasonable request.
